# Another Record: Ocean Warming Continues through 2021 despite La Niña Conditions

**DOI:** 10.1007/s00376-022-1461-3

**Published:** 2022-01-11

**Authors:** Lijing Cheng, John Abraham, Kevin E. Trenberth, John Fasullo, Tim Boyer, Michael E. Mann, Jiang Zhu, Fan Wang, Ricardo Locarnini, Yuanlong Li, Bin Zhang, Zhetao Tan, Fujiang Yu, Liying Wan, Xingrong Chen, Xiangzhou Song, Yulong Liu, Franco Reseghetti, Simona Simoncelli, Viktor Gouretski, Gengxin Chen, Alexey Mishonov, Jim Reagan

**Affiliations:** 1 International Center for Climate and Environment Sciences, Institute of Atmospheric Physics, Chinese Academy of Sciences, Beijing, 100029 China; 2 Center for Ocean Mega-Science, Chinese Academy of Sciences, Qingdao, 266071 China; 3 University of St. Thomas, School of Engineering, Minnesota, 55105 USA; 4 National Center for Atmospheric Research, Boulder, Colorado 80307 USA; 5 National Oceanic and Atmospheric Administration, National Centers for Environmental Information, Silver Spring, Maryland 20910 USA; 6 Department of Meteorology & Atmospheric Science, The Pennsylvania State University, University Park, Pennsylvania 16802 USA; 7 Institute of Oceanology, Chinese Academy of Sciences, Qingdao, 266071 China; 8 National Marine Environmental Forecasting Center, Ministry of Natural Resources of China, Beijing, 100081 China; 9 College of Oceanography, Hohai University, Nanjing, 210098 China; 10 National Marine Data and Information Service, Tianjin, 300171 China; 11 Italian National Agency for New Technologies, Energy and Sustainable Economic Development, S. Teresa Research Center, Lerici, 19032 Italy; 12 Istituto Nazionale di Geofisica e Vulcanologia, Sede di Bologna, Bologna, 40128 Italy; 13 South China Sea Institute of Oceanology, Chinese Academy of Sciences, Guangzhou, 510301 China; 14 ESSIC/CISESS-MD, University of Maryland, College Park, MD 20742 USA

**Keywords:** La Niña, ocean heat, ocean warming, attribution, observation, 拉尼娜, 海洋热含量, 海洋变暖, 归因, 观测

## Abstract

The increased concentration of greenhouse gases in the atmosphere from human activities traps heat within the climate system and increases ocean heat content (OHC). Here, we provide the first analysis of recent OHC changes through 2021 from two international groups. The world ocean, in 2021, was the hottest ever recorded by humans, and the 2021 annual OHC value is even higher than last year’s record value by 14 ± 11 ZJ (1 zetta J = 10^21^ J) using the IAP/CAS dataset and by 16 ± 10 ZJ using NCEI/NOAA dataset. The long-term ocean warming is larger in the Atlantic and Southern Oceans than in other regions and is mainly attributed, via climate model simulations, to an increase in anthropogenic greenhouse gas concentrations. The year-to-year variation of OHC is primarily tied to the El Niño-Southern Oscillation (ENSO). In the seven maritime domains of the Indian, Tropical Atlantic, North Atlantic, Northwest Pacific, North Pacific, Southern oceans, and the Mediterranean Sea, robust warming is observed but with distinct inter-annual to decadal variability. Four out of seven domains showed record-high heat content in 2021. The anomalous global and regional ocean warming established in this study should be incorporated into climate risk assessments, adaptation, and mitigation.

## Abstract (zh)

人类活动导致大气中温室气体的浓度上升,造成了地球系统的净热量吸收和海洋热含量增加。本文发布了两个国际机构的2021年海洋热含量数据,数据表明:2021年海洋升温持续——成为有现代海洋观测记录以来海洋最暖的一年。相对于2020年,2021年全球海洋上层2000米热含量上升了14 ± 11 ZJ (1 zetta J = 10^21^ J)(IAP/CAS数据)、以及16 ± 10 ZJ(NOAA/NCEI数据)。海洋长期变暖趋势在南大洋、中低纬度大西洋区域更强,地球系统模式的单个因子强迫实验证明,温室气体增加是主要的驱动因子;而年际尺度的海洋热含量变化主要受到厄尔尼诺-南方涛动模态调控。此外,本文给出了全球7个主要海域的海洋变暖测算,发现地中海、北大西洋、南大洋、北太平洋海区温度均创历史新高。最后,本文提出需要充分将全球和区域海洋变暖的影响纳入气候风险评估、气候变化影响和应对当中。

**Article Highlights:**

- The world ocean, in 2021, was the hottest ever recorded by humans.
- The warming pattern is mainly attributed to increased anthropogenic greenhouse gas concentrations, offset by the impact of aerosols.
- Ocean warming has far-reaching consequences and should be incorporated into climate risk assessments, adaptation, and mitigation.

## 1. Introduction

The increased concentrations of greenhouse gases in the atmosphere from human activities trap heat within the climate system and result in massive changes in the climate system. As a result, outgoing energy from the Earth system is not balancing the incoming solar radiation, thus creating Earth's Energy Imbalance (EEI) in the climate system (Trenberth et al., 2014; von Schuckmann et al., 2016a, 2020a; Wijffels et al., 2016; Johnson et al., 2018; Cheng et al., 2019a). The oceans store over 90% of EEI, leading to an increase of ocean heat content (OHC), which currently provides the best estimate for EEI (Hansen et al., 2011; IPCC, 2013; Rhein et al., 2013; Trenberth et al., 2016; Abram et al., 2019). Ocean warming leads to increased ocean vertical stratification, thermal expansion, and sea-level rise. These processes provide a compelling means to quantify climate change (Cheng et al., 2018).

This study provides the first analysis of recent OHC changes through 2021. Two international data products include those from the Institute of Atmospheric Physics (IAP) at the Chinese Academy of Sciences (CAS) (Cheng et al., 2017) and the National Centers for Environmental Information (NCEI) of the National Oceanic and Atmospheric Administration (NOAA) (Levitus et al., 2012). Both datasets corrected systematic errors and then used thorough mapping methods to convert discrete ocean measurements into a comprehensive picture of the ocean. Both global and regional analyses of OHC changes are provided in this study.

## 2. Data and Methods

The IAP/CAS and NCEI/NOAA analyses are based on available *in situ* observations from various measurement devices held in the World Ocean Database (WOD) of the NCEI/NOAA. Data from all instruments are used, including eXpendable BathyThermographs (XBTs), profiling floats from Argo, moorings, gliders, Conductivity/Temperature/Depth devices (CTDs), bottles, and instruments on marine mammals (Boyer et al., 2018). The XBT biases are corrected according to Cheng et al. (2014) for IAP/CAS and Levitus et al. (2009) for NCEI/NOAA. Model simulations guide the mapping method from point measurements to the comprehensive grid in the IAP/CAS product. At the same time, sampling errors are estimated by sub-sampling the Argo data at the locations of the earlier observations (a full description of the method is in Cheng et al., 2017).

The Argo Program is part of the Global Ocean Observing System. The Argo observing network achieved a near-global upper-2000 m coverage since about 2005 (Argo, 2020). Argo data are made freely available by the International Argo Program and the contributing national programs (http://www.argo.ucsd.edu; http://argo.jcommops.org).

In addition to these observations, the Community Earth System Model Version 1 (CESM1) Large Ensemble (LENS; Kay et al., 2015) data are used to explore the influence of different forcings (aerosols, greenhouse gasses, industrial aerosols, biomass aerosols, land use, and land cover) on the formation of OHC patterns in a large ensemble simulation of climate. Previous assessments indicate that CESM is one of the most skilled models representing the global energy budget, water cycles, and associated dynamics (Fasullo, 2020).

## 3. Global ocean changes

The up-to-date data indicate that the OHC in the upper 2000 m layer of the world's oceans has increased with a mean rate of 5.7 ± 1.0 ZJ $yr^{-1}$ for the 1958–2021 period (IAP/CAS) and 4.7 ± 1.0 ZJ $yr^{-1}$ for the 1958–2021 period (NCEI/NOAA) (Fig. 1a). Both datasets show an unambiguous increase in ocean warming since the late 1980s (Fig. 1a). From 1986–2021, the average annual increase is 9.1 ± 0.3 ZJ $yr^{-1}$ for IAP and 8.3 ± 0.7 ZJ $yr^{-1}$ for NCEI/NOAA. These warming rates represent a maximum of 8-fold increase compared to 1958–85 (1.2 ± 0.6 ZJ $yr^{-1}$ IAP/CAS, 1.5 ± 1.0 ZJ $yr^{-1}$ for NCEI/NOAA). Moreover, each decade since 1958 has been warmer than the preceding decades.

The 2021 annual OHC value is higher than the last year's value, by 14 ± 11 ZJ using the IAP/CAS data and by 16 ± 10 ZJ using NCEI/NOAA (95% confidence interval). Both projections are the highest on record (Table 1). Differences between the OHC analyses reflect the uncertainties in the calculation due to data quality, mapping differences, and data coverage. Nevertheless, it is evident that according to both IAP/CAS and NCEI/NOAA, the 2021 oceans were the hottest ever recorded by humans.

There are notable inter-annual fluctuations in the OHC record (Fig. 1b); Cheng et al. (2018) indicated that it requires ~four years for the long-term trend signal to significantly exceed the inter-annual variability at the 95% confidence interval, noting that ENSO is the dominant driver of year-to-year variations in OHC (i.e., Cheng et al., 2019b).

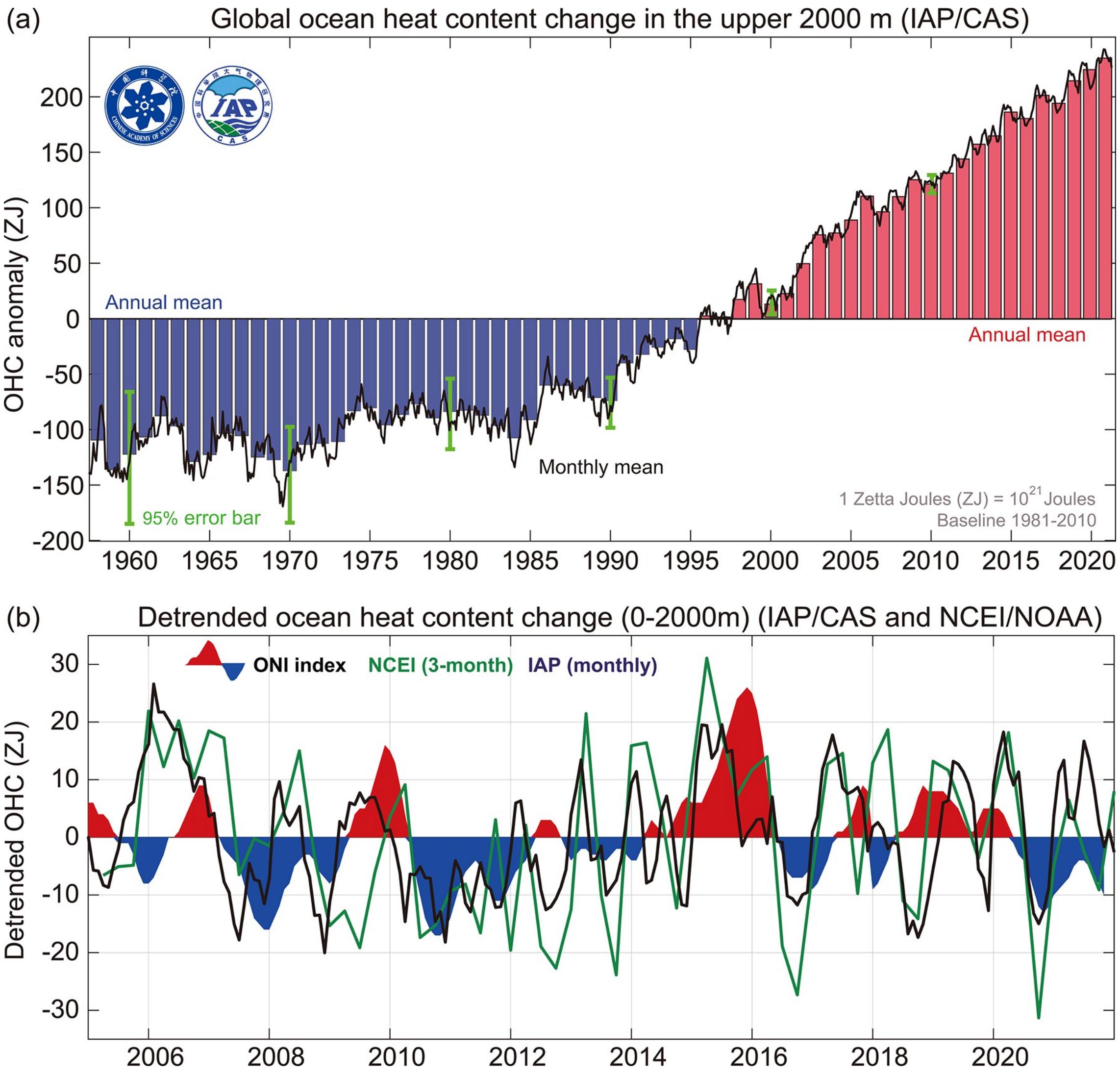

**Fig. 1**. (upper) The global upper 2000 m OHC from 1958 through 2021. The histogram presents annual anomalies relative to a 1981–2010 baseline, with positive anomalies shown as red bars and negative anomalies as blue. Units: ZJ. (bottom) Detrended OHC time series from 2005 to 2021 compared with ONI index.

**Table 1**. Ranked order of the hottest five years of the global ocean, since 1955. The OHC values are anomalies for the upper 2000 m in units of ZJ relative to the 1981–2010 average.

| Rank | Year | IAP/CAS | NCEI/NOAA |
|---|---|---|---|
| 1 | 2021 | 235 | 227 |
| 2 | 2020 | 221 | 211 |
| 3 | 2019 | 214 | 210 |
| 4 | 2017 | 202 | 189 |
| 5 | 2018 | 195 | 196 |

The Indo-Pacific basin transitioned from a weak El Niño state in the first six months of 2020 toward a La Niña state during the last half of 2020, and a La Niña state has since continued in 2021. There is a decreasing tendency in OHC during the transition from El Niño to La Niña because of ocean heat release associated with anomalous warming of sea surface conditions in the tropical Pacific Ocean (Cheng et al., 2019b) (Fig. 1b). During La Niña, the ocean absorbs heat, which caused an increase in OHC from late-2020 to early 2021 (Fig. 1b). Similar OHC changes also have occurred during the 2009, 2015, and 2017 El Niño events. The El Niño-driven heat buildup and higher sea levels are most typical of the tropical western Pacific region (see Fig. 2).

In addition to providing an update on the 2021 OHC, we present an improved calculation for previous years. Both IAP/CAS and NCEI/NOAA have re-calculated the 2020 OHC values using the most up-to-date observations. A notable revision of 0–2000 m OHC anomaly from 234 ZJ to 221 ZJ is found for IAP/CAS data. With this correction, the IAP/CAS and NCEI/NOAA OHC time series become consistent in 2020 and 2021 (Fig. 1b), increasing confidence in the near real-time OHC calculation. By comparing the two versions of WOD in situ observational data, it was found that data Quality-Control (QC) flags had not been assigned in the previous dataset (i.e., there is no QC applied to the

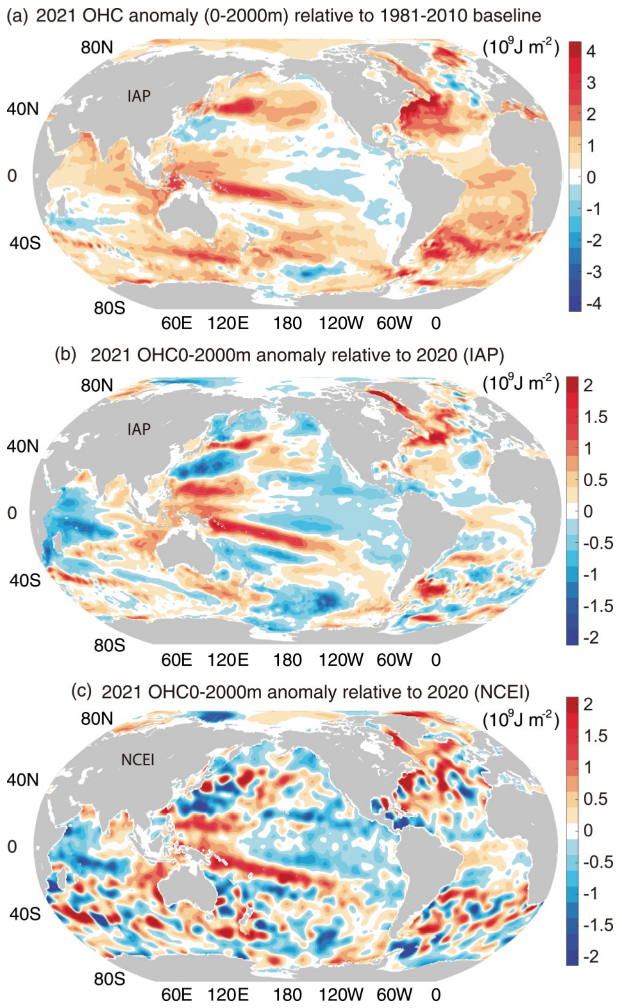

**Fig. 2**. (a) The ocean heat content anomaly in 2021 relative to 1981–2010 baseline. (b) The difference of OHC for upper 2000 m between 2021 and 2020. (c) As in (b) but for NCEI/NOAA data. Units: $10^9$ J m$^{-2}$. [Data updated from Cheng et al. (2017) in (a) and (b), from Levitus et al. (2012) in (c)].

data), highlighting the importance of QC in OHC calculations. Considering the non-negligible impact of QC on the OHC estimate, comprehensive quantification of QC uncertainty continues to be a priority.

## 4. Regional patterns of ocean warming

The spatial pattern of upper OHC anomaly (0–2000 m) (Fig. 2a) illustrates several key features, including an anomaly maxima near 40° in both hemispheres and broad anomalous warming in the Atlantic Ocean relative to other ocean basins, but a pronounced minimum in the northern North Atlantic Ocean. The drivers of these features can be explored by using so-called single-forcing large ensembles, within which only a single external climate forcing agent is held fixed over time in a multi-member ensemble experiment (Deser et al., 2020). By averaging across ensemble members and differencing with the fully-forced ensemble, the contributions of internal variability are reduced to allow direct estimation of the response to forcing. Important uncertainties exist in inferring natural changes from such ensembles, including uncertainties related to model structure and imposed external forcing agents. Strategies for addressing these uncertainties are addressed below.

Here, we use the Community Earth System Version 1 (CESM1) Large Ensemble (CESM1-LE; Kay et al., 2015) and Single-Forcing Large Ensemble (CESM1-SF; Deser et al., 2020) to attribute the drivers of observed OHC trend patterns from 1979 to 2020 (Fig. 3). A strong correlation pattern exists between the fully-forced ensemble mean and observed changes (Fig. 3a vs. Fig. 2a), thus providing evidentiary support for both the fidelity of the CESM1-LE and the emergence of forced changes in the presence of internal variability (i.e., Cheng et al., 2018; Fasullo and Nerem, 2018). Many of the salient features apparent in observations are also evident in the ensemble mean, suggesting a role for forcing in their development. Examples include the relative maxima near 40° in each hemisphere, the region of strong cooling in the northern Atlantic southeast of Greenland, the subtle changes in the Pacific Ocean equatorward of 30°, and the relative warming of the Atlantic Ocean as compared to other ocean basins (Fig. 3a). Variability in trends across ensemble members is largest in the oceans north of 30°N (Fig. 3b).

The influence of industrial aerosols (AER, Fig. 3c) is strong and pervasive across much of the global ocean, exerting a strong cooling influence between 30°N and 60°S and significantly contributing to the regional cooling southeast of Greenland. The influence of biomass aerosols (BMB, Fig. 3d) is more regional yet important, with strong cooling signals evident in all basins and a warming contribution to the North Atlantic. The contribution of greenhouse gases (GHG, Fig. 3e) is notable in nearly all regions, with substantial warming, especially near 40°S and across the Atlantic Ocean. A strong contribution to cooling southeast of Greenland is also evident. Lastly, land use and land cover (LULC) contributions are small, with significant areas spanning less than 5% of the global ocean.

Together these results promote an understanding of the observed pattern of ocean warming since 1980. Forcing from greenhouse gases dominates many of the observed features but contributions from AER and BMB are also important. Pervasive warming of the global ocean is driven by GHG and offset somewhat by both AER and BMB (Fig. 3c, d). The elevated warming of the Atlantic Ocean relative to other basins results from both the elevated warming effects of GHG and the reduced cooling effects of AER in the Atlantic. Together these effects are offset somewhat by a strong regional cooling driven by BMB. The region of strong cooling southeast of Greenland is driven by both GHG and AER influences (associated with AMOC changes). It stands out as one of the few regions where GHG and AER effects mutually reinforce each other. Lastly, the relative minimum in the tropical Pacific Ocean equatorward of 30° results from the combined influences of relatively weak GHG-driven warming and the offsetting effects of both AER and BMB.

This diagnosis of the drivers of OHC trend patterns relies on the fidelity of the CESM1 and the estimates of external forcing agents used in the CESM1-LE. Further exploration with alternative models and forcings will play an important role in establishing confidence in the interpretations based on the experiments used here. Because of space limitations here, accounting for the uncertainties in forcings (Fasullo et al., 2021; Fyfe et al., 2021; Smith and Foster, 2021) remains a topic for future work. Formal detection and attribution analysis plus single forcing experiments with more climate models are also being conducted.

Comparing the spatial OHC anomalies in 2021 versus 2020 shows an imprint of La Niña (Figs. 2b, c, Cheng et al., 2019b). The NCEI/NOAA data (Fig. 2c) shows a consistent spatial pattern compared with IAP/CAS (Fig. 2b), but the anomalies are spottier and tied to the spatial covariance in mapping strategy. The IAP/CAS product is smoother than NCEI/NOAA because of the stronger effect of smoothing (Cheng et al., 2017). There is a strong increase of OHC in the western Pacific that extends into the South and North Pacific regions to 20°S and 20°N, and a broad decrease of OHC in the eastern Pacific, including coastal Peru and the California oceans (Figs. 2b, c). A reverse zonal gradient (warm in the east and cold in the west) is evident in the Indian Ocean. This pattern in the Indo-Pacific Ocean mainly reflects the thermocline changes during ENSO: the thermocline is steeper during La Niña, with a deeper thermocline in the western Pacific-Indonesian warm pool. Meanwhile, the thermocline uplifts in the tropical eastern Pacific and western Indian oceans, leading to cooling anomalies. The tropical Atlantic Ocean shows a small decrease of OHC (20°S–20°N), likely due to two separate but reinforcing factors: (1) the shift of the Walker circulation during La Niña events increases evaporation in the tropical Atlantic (Wang, 2019) and (2) those same La Niña conditions contributed to an especially active and energetic Atlantic hurricane season as tropical storm-related mixing leads to substantial

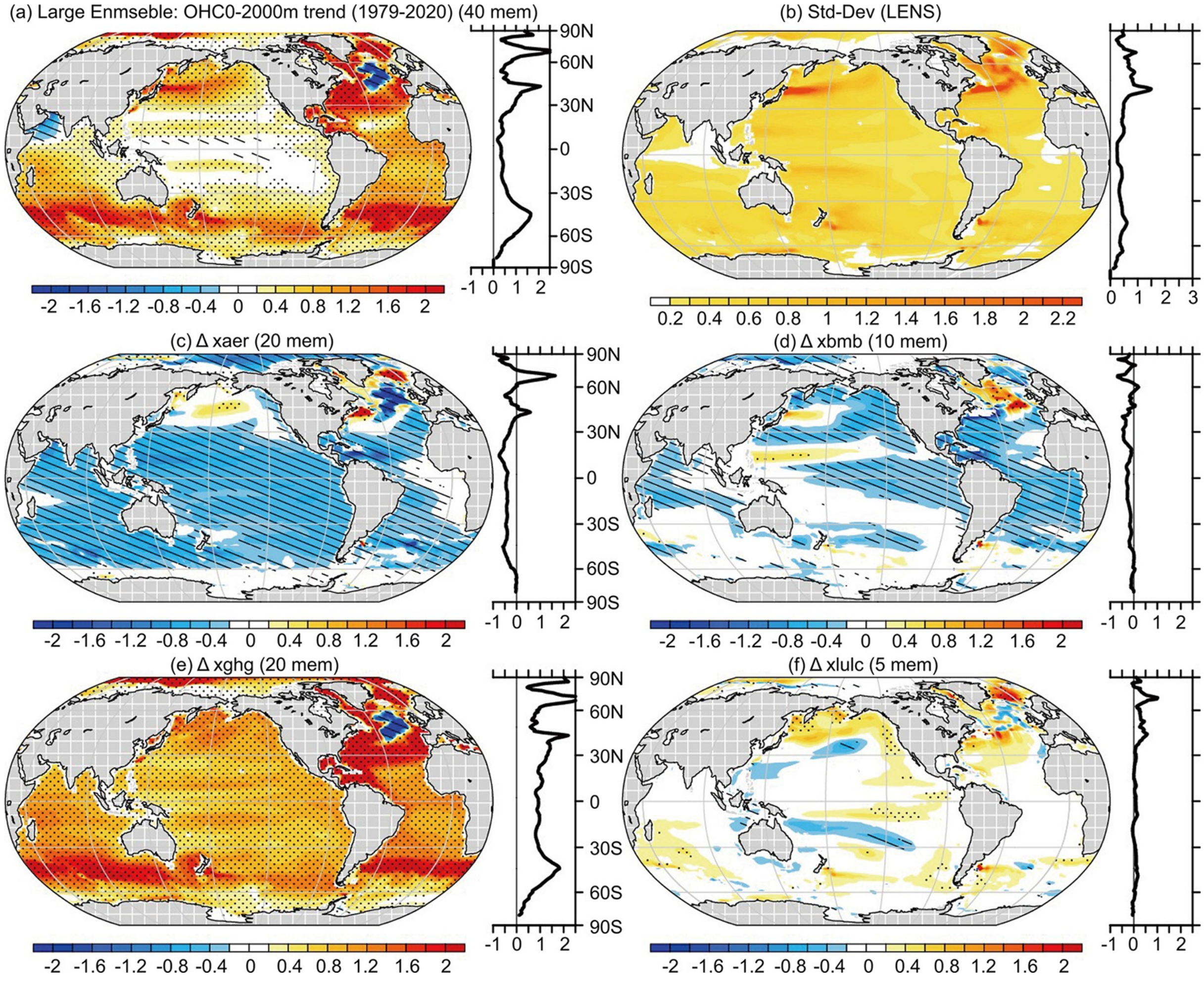

**Fig. 3**. Ensemble mean trends in 0–2000 m OHC in (a) the CESM1-LE and CESM1-SF estimated contributions from (c) industrial and (d) biomass aerosols, (e) greenhouse gasses, and (f) change in land use and land cover. Units: $10^9$ J m$^{-2}$. Stippled and hatched regions correspond to areas where positive and negative trends exceed twice the standard ensemble error, respectively. (b) The standard deviation of CESM1-LE members in 0–2000 m OHC trend.

poleward ocean heat export from the tropics (Sriver and Huber, 2007).

## 5. Basin-wide changes and regional hot spots

A regional assessment of ocean warming is relevant to community risk assessment and societal adaptation, even more so than global metrics. Here, we briefly assess basin OHC changes, including several regional hot spots.

### 5.1. *The northwest Pacific Ocean*

The Northwest Pacific Ocean is the most active basin for tropical storms on the planet, accounting for one-third of all tropical cyclones. Changes in its nature are directly relevant to weather conditions and societies in East Asia. The OHC shows strong inter-annual and decadal fluctuations in this area (Fig. 4a), which are predominantly associated with internal climate variabilities such as ENSO and Pacific Decadal Variability (PDV) (Cheng et al., 2019b; Xiao et al., 2020). The typhoon activities in the Northwest Pacific show correlations with PDV and further reveal the remote impacts of the tropical Indian Ocean and Western Pacific (Yang et al., 2018). Nevertheless, the mean OHC over the recent decade (2012–21) is higher than the 1981–2010 average, which could, in turn, impact the regional OHC (Wang et al., 2014).

### 5.2. *The Indian Ocean*

There was no significant trend in the Indian OHC during the late 20$^{th}$ century (Fig. 4b), owing to interdecadal changes of the Indo-Pacific Walker circulation that drove an upper-layer cooling of the tropical Indian Ocean and greatly moderated the greenhouse-gas forced warming (Ummenhofer et al., 2021). Unprecedented rapid warming during the 2000s was mainly associated with an increased Indonesian throughflow heat transport under a negative Interdecadal Pacific Oscillation (IPO) condition (La Niña-like) of the tropical Pacific (Lee et al., 2015; Li et al., 2018). The warming trend has been delayed since ~2013, as the IPO shifted back to its positive phase. Hence, the pronounced interannual fluctuations in the past decade were primarily dictated by ENSO. The thermocline of the southern tropical Indian Ocean is particularly sensitive to teleconnection imprints of ENSO (Xie et al., 2002), as evidenced by the thermocline depression and OHC increase during the 2015−16 super El Niño and subsequent thermocline shoaling and OHC decrease during the 2017−18 La Niña (Li et al., 2020; Volkov et al., 2020) (Fig. 4b). The climate of the Southern Ocean also modulates the subtropical sector of the southern Indian Ocean. During past decades, the poleward migration of westerly winds has led to a southward shift of the subtropical circulation gyre and thereby caused a multi-decadal increasing trend in the OHC of the subtropical southern Indian Ocean (Yang et al., 2020; Duan et al., 2021).

### 5.3. *The tropical Atlantic Ocean*

The tropical western North Atlantic Ocean 10°–30°N, where hurricanes initiate and develop, has shown a continuous increase of OHC since 1958 (Fig. 4c; Trenberth et al., 2018). La Niña conditions typically are associated with reduced tropical storm activity in the eastern tropical Pacific and a greater risk of enhanced activity in the tropical North Atlantic. In addition to the global warming signature from continuous unabated warming since 1958 (Fig. 4c), this is associated with high levels of Atlantic tropical storm activity during the past two northern summers (Emanuel, 2021a, b). During 2021 there were 21 named storms, ~30% fewer than the record 30 named storms of 2020. When atmospheric conditions are favorable, the increased thermal energy provided by high OHC fuels increased tropical storm activity (in terms of both frequency, intensity, size and/or lifetime) and also increases rainfall (Trenberth et al., 2018).

### 5.4. *The Mediterranean Sea*

In 2021, the OHC in the Mediterranean Sea was the highest on record since reliable records exist (Fig. 4d). A marked temperature increase appeared in the last few decades, starting from the Eastern Basin where warmer Intermediate Waters (IW) formed and spread towards the Western Basin on their way back to the North Atlantic (Pinardi et al., 2015; Section 3.1 in von Schuckmann et al., 2016b; Simoncelli et al., 2018, 2019; Storto et al., 2019). The Sicily Channel represents the chokepoint between the Eastern and Western Mediterranean basins where a two-layer system brings Atlantic Waters (0–150 m) eastwards and IW (150–450 m) westwards (Schroeder et al., 2017). A temperature and salinity time series collected since 1993 at 400 m depth at the Sicily Channel show increasing trends for both parameters, stronger than those observed at intermediate depths in different regions of the global ocean (Ben Ismail et al., 2021). The reported IW rate of temperature increase averages 0.026°C yr$^{-1}$ with a maximum value of 0.034°C yr$^{-1}$ after 2011. The IW enters the Tyrrhenian Sea after crossing the Sicily Channel, where the temperature has been monitored with XBT probes since 1999 along the MX04 Genova-Palermo transect (Fig. 5d) as part of the Ship of Opportunity program of GOOS. Figure 5 presents two Hovmöller plots of mean temperature anomalies in the layer 0–800 m layer computed along the transect considering different baselines: one subtracting the monthly average computed from the MX04 survey (Fig. 5a); the other subtracting the IAP/CAS baseline 1981–2010 (Fig. 5b). Consistent and continuous warming of the IW appears from spring 2013 in the 150–450 m layer and propagates progressively deeper. The mean temperature evolution in the 150–450 m layer (Fig. 5c) indicates a steep increase between 2014 and 2017 and a linear trend over the time period 1999–2021 equal to 0.028°C yr$^{-1}$, consistent with the one observed in the Sicily Channel. The overall mean temperature variation is approximately 0.6°C, with temperatures ranging from 13.8°C to 14.4°C. The warm IW propagated northwards mainly from 38° to 42°N and, as in the Sicily Channel, the IW exhibited a slight decrease in 2018-2019. The data gathered in September and

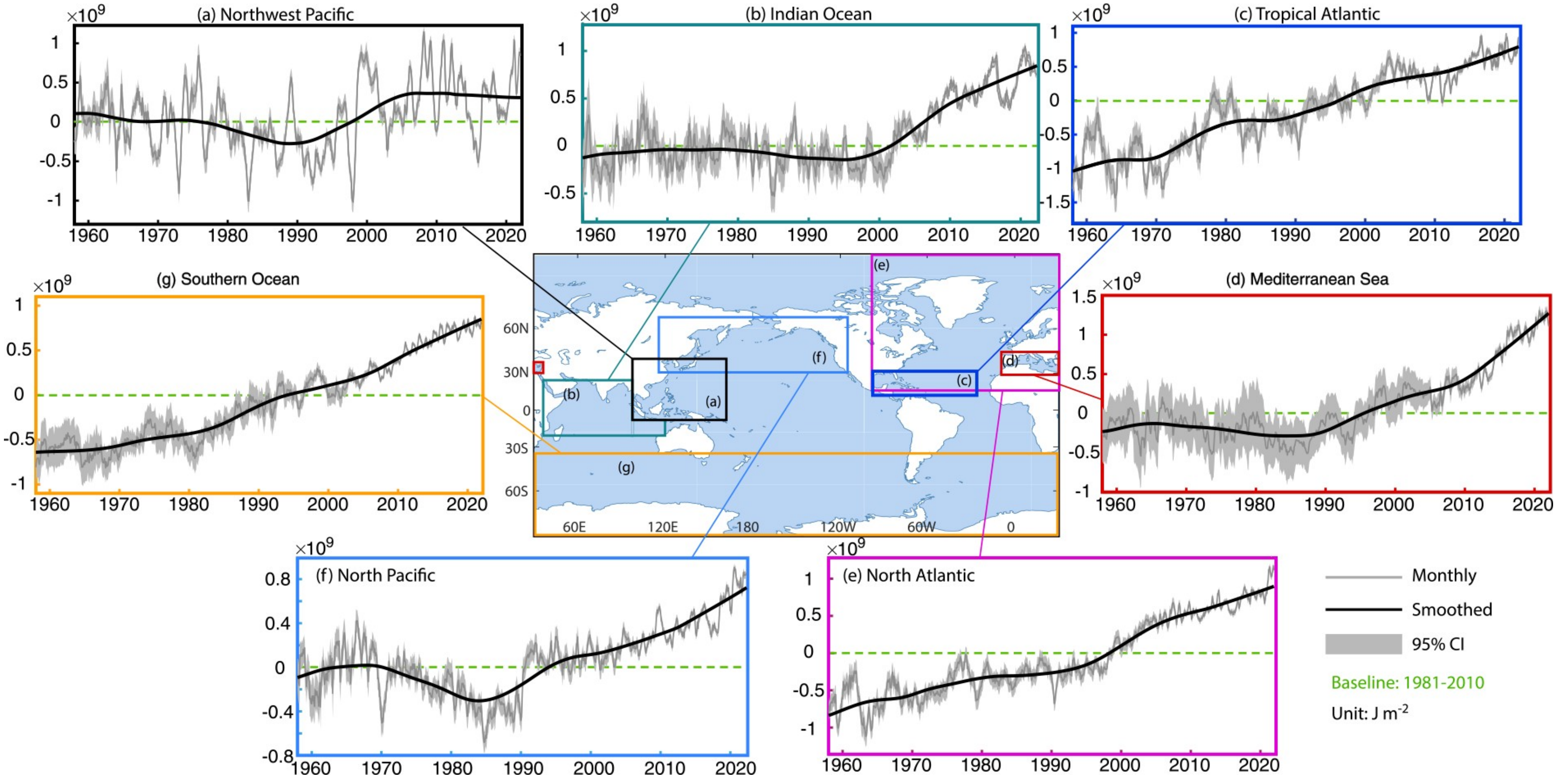

**Fig. 4**. Regional observed upper 2000 m OHC change from 1955 through 2021 relative to 1981–2010 baseline. The time series (black) are smoothed by LOWESS (locally weighted scatterplot smoothing) with a span width of 24 months. The gray shadings are the 95% confidence interval. [Data updated from Cheng et al. (2017)].

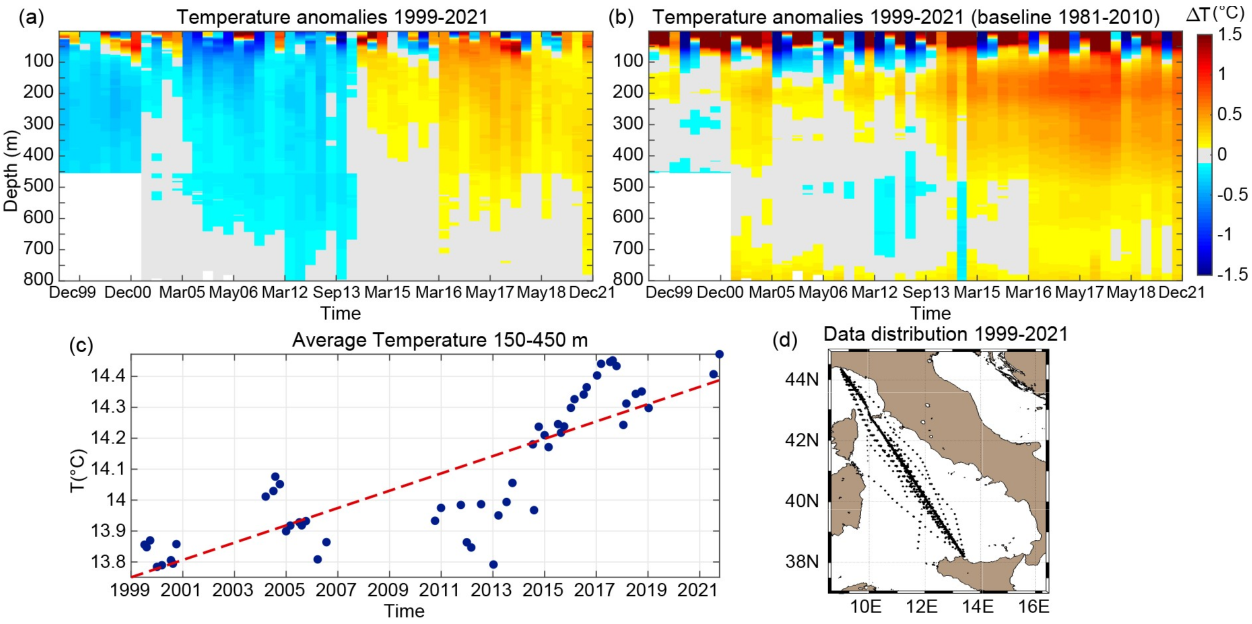

**Fig. 5**. The temperature along the MX04 Genova-Palermo transect in the 1999–2021 time period recorded by XBT probes from ships of opportunity: (a) Hovmöller plot of mean temperature anomalies computed subtracting the overall average 1999–2021 from each monitoring survey; (b) Hovmöller plot of mean temperature anomalies computed subtracting the IAP/CAS 1981–2010 baseline from each monitoring survey; (c) mean temperature values and relative linear trend in the layer 150–450 m; (d) data distribution map in the Tyrrhenian and Ligurian Seas (Western Mediterranean).

December 2021 report mean values greater than or equal to 14.4°C, as in 2017, but the heat penetrates progressively deeper and occupies a larger area.

### 5.5. *The North Atlantic Ocean*

The North Atlantic Ocean shows a warming trend for the entire instrumental record, with an increasing trend commencing around 2000 (Fig. 4e). The most dramatic warming occurs in the Gulf Stream region (Figs. 2, 3). Despite the broad-scale warming, a key feature of the North Atlantic Ocean is a cooling region south of Greenland (the so-called "warming hole") (Seidov et al., 2017). In single forcing experiments, it is demonstrated that both GHG and AER forcings can produce such a cooling signature (Fig. 3). Previous studies have indicated that this cooling trend reveals a slowdown of the Atlantic meridional overturning circulation, which leads to reduced oceanic heat transport into the warming hole region (Rahmstorf et al., 2015; Keil et al., 2020). Other potential culprits include the changes to the Gulf Stream extension region (Seidov et al., 2019; Piecuch, 2020; Boers, 2021), local atmospheric forcing (Li et al., 2021), and Indian Ocean warming (Hu and Fedorov, 2020). In 2021, the North Atlantic OHC was the highest on record since 1958 (Fig. 4e).

### 5.6. *The North Pacific Ocean*

The North Pacific (30°–62°N) showed a dramatic increase in OHC after 1990 and reached a record high level in 2021 (Fig. 4f). The North Pacific Ocean warming is broad and deep-reaching. Warming anomalies in 2021 (relative to the 1981–2010 baseline) are ~2°C near the surface and 1°C at ~300 m in the middle North Pacific (Fig. 6). The relentless increases in OHC have direct implications for the frequency, intensity, and extent of marine heat waves (MHWs) and other "hot spots" within the ocean (Holbrook et al., 2019). A series of MHWs have developed here within the past decade. These are typically triggered by benign atmospheric conditions with anticyclonic circulation, decreased evaporative cooling, and lighter winds that lead to less ocean mixing (Holbrook et al., 2019). In the North Pacific winter, such conditions are favored by La Niña through an atmospheric bridge (Trenberth et al., 1998), and this helped set up the North Pacific MHW in 2013-14. Such events impact marine life and propagate through the food web to larger marine creatures. "The Blob" was one such strong event from 2014-16 and demonstrated that the entire ocean food web can be severely impacted due to these extremes (Cornwall, 2019). The 2021 North Pacific warm "blob" was a continuation of the 2020 anomaly. Scannell et al. (2020) indicate that the oceanic conditions (OHC, salinity, and stratification) support the development of marine heat waves in the North Pacific Ocean. The marine heat wave is also associated with an atmospheric circulation pattern. A "high-pressure heat dome" developed in late June over the Pacific Northwest and southwest Canada, along with temperatures that exceeded 40°C (a record-breaking major heatwave), which resulted in widespread wildfires and associated destruction. In September and October 2021, the MHW continued and was strongest in the central North Pacific, consistent with La Niña influences through the atmospheric bridge from the

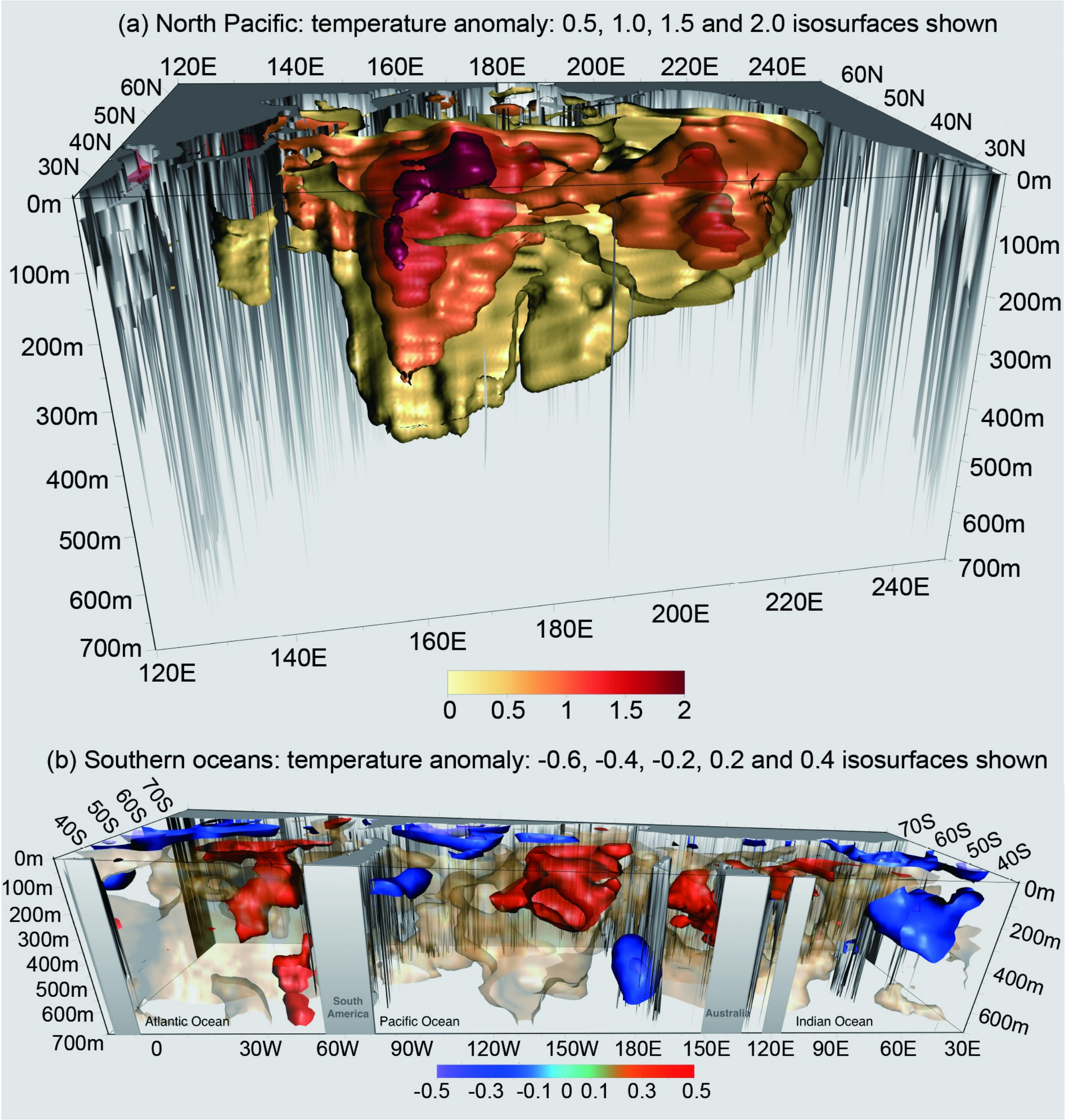

**Fig. 6**. 3-D fields of oceanic temperature changes at 2021 (January to September) relative to 1981–2010 baseline in the North Pacific Ocean (30°–62°N) (upper), and in the Southern oceans (78°–30°S) (bottom). The IAP/CAS data are used, and the illustration is modified from Seidov et al. (2021).

tropics.

### 5.7. *The Southern Ocean*

According to model simulations, the Southern Ocean (SO) served as a major anthropogenic heat sink in the past century (Frölicher et al., 2015); observation-based estimates suggest that the SO accounted for 30%–60% of the global OHC increase since ~1970 (IPCC, 2019), contemporaneous with significant ocean warming (Fig. 4g; Gille, 2002). Mass subduction of water from the SO mixed layer serves as a rapid pathway for anthropogenic warming signatures to be communicated to the deep ocean (Marshall and Speer, 2012). Based on CMIP5 model simulations, Silvy et al. (2020) showed that the anthropogenic signal 'emerges' from the noise and overwhelms the natural variability much more quickly in the well-ventilated SO water masses than in Northern Hemisphere waters. The SO shows a clear warming peak on the northern flank of the Antarctic Circumpolar Current (ACC) (Figs. 2 and 6) (Böning et al., 2008; Gouretski et al., 2013; Roemmich et al., 2015), corresponding to the rapid warming of the Subantarctic Mode Water (SAMW) (Gao et al., 2018) and Antarctic Intermediate Water (Schmidtko and Johnson, 2012) with widespread impacts across the entire Southern Hemisphere. By 2021, the warming exceeded 0.6°C in the major formation regions of the SAMW, compared to the 1981–2010 baseline (Fig. 6b). The Antarctic continental shelf and adjacent seas also show significant warming trends (Schmidtko et al., 2014) linked to the detected warming of abyssal and bottom waters over the globe (Purkey and Johnson, 2010, 2013). The surface layer of the subpolar regions shows little warming and has slightly cooled over the past decades, owing to wind-driven upwelling, increased northward heat export to the northern flank of the ACC, and sea ice changes (Armour et al., 2016). Purich et al. (2018) added that the freshening of the SO — linked to the amplification of the global hydrological cycle — may also favor subpolar surface cooling by reducing the entrainment of subsurface warm water. These complex heat uptake and storage patterns are primarily attributable to greenhouse-gas forcing, with stratospheric ozone depletion playing a secondary role (Swart et al., 2018).

## 6. Summary and implications for climate actions

This study provides up-to-date estimates of OHC through 2021 based on two international datasets. Single forcing simulations are used to analyze the contribution of different forcings on the regional OHC pattern. The regional and global changes both reveal a robust and significant ocean warming since the late-1950s — the entirety of the reliable instrumental record. Natural variability and change in ocean circulation play important roles locally, but the predominant changes result from human-related changes in atmospheric composition. As oceans warm, the water expands, and sea level rises. Preparing for sea level rise and its implications for coastal communities are particularly important.

Changes to engineering design, building codes, and modifications to coastal development plans are recommended in anticipation of increased sea levels and increases in extreme precipitation events, which are already being observed (Abraham et al., 2015, 2017; Scambos and Abraham, 2015). In addition, warmer oceans supercharge the weather systems, creating more powerful storms and hurricanes, and increased precipitation. Warmer oceans lead to a warmer and moister atmosphere that promotes more intense rainfall in all storms, especially hurricanes, thereby increasing the risk of flooding (Trenberth et al. 2003; IPCC, 2021). Warming ocean waters threaten marine ecosystems and human livelihoods, for example, coral reefs and fisheries (IPCC, 2019).

The United Nations has proposed 17 inter-linked goals and 169 targets focused on maintaining the health and vitality of human societies and natural ecosystems worldwide (UN, 2021). The Sustainable Development Goals (SDGs) were set up in 2015 by the United Nations General Assembly and are intended to be achieved by 2030. Many goals are closely related to the health of the world's oceans, and the ocean's health is vital to the SDGs. For example, the SDG13-Climate Actions; SDG14-Life Below Water; SDG6-Clean Water and Sanitation; SDG2-Zero Hunger; SDG3-Good Health and Well-Being (Abram et al., 2019; von Schuckmann et al., 2020) are all either directly or indirectly related to oceans. In 2021, the United Nations initiated the Decade of Ocean Science for SDGs to increase the awareness of many problems the ocean faces and to build up capability for ocean monitoring and scientific research. This study indicates that there are still uncertainties and knowledge gaps in monitoring ocean warming; for example, the quantification of uncertainty from inter-annual to multidecadal scales and the impact of data QC and regional differences of OHCs revealed by different datasets. Thus, better awareness and understanding of ocean dynamics are fundamental in combating climate change.

***Acknowledgements***. The IAP/CAS analysis is supported by the National Natural Science Foundation of China (Grant No. 42122046, 42076202), Strategic Priority Research Program of the Chinese Academy of Sciences (Grant No. XDB42040402), National Natural Science Foundation of China (Grant No. 42076202), National Key R&D Program of China (Grant No. 2017YFA0603202), and Key Deployment Project of Centre for Ocean Mega-Research of Science, CAS (Grant Nos. COMS2019Q01 and COMS2019Q07). NCAR is sponsored by the US National Science Foundation. The efforts of Dr. Fasullo in this work were supported by NASA Award 80NSSC17K0565, and by the Regional and Global Model Analysis (RGMA) component of the Earth and Environmental System Modeling Program of the U.S. Department of Energy's Office of Biological & Environmental Research (BER) via National Science Foundation IA 1844590. The efforts of Dr. Mishonov and Mr. Reagan were partially supported by NOAA (Grant NA14NES4320003 to CISESS-MD at the University of Maryland). The IAP/CAS data are available at http://www.ocean.iap.ac.cn/ and https://msdc.qdio.ac.cn/. The NCEI/NOAA data are available at https://www.ncei.noaa.gov/products/climate-data-records/global-ocean-heat-content. The historical XBT data along the MX04 line (Genova-Palermo) are available through SeaDataNet - Pan-European infrastructure (http://www.seadatanet.org) for ocean and marine data management. Since 2021, XBT data have been collected in the framework of the MACMAP project funded by the Istituto Nazionale di Geofisica e Vulcanologia in agreement between INGV, ENEA, and GNV SpA shipping company that provides hospitality on their commercial vessels.

***Open Access*** This article is licensed under a Creative Commons Attribution 4.0 International License, which permits use, sharing, adaptation, distribution and reproduction in any medium or format, as long as you give appropriate credit to the original author (s) and the source, provide a link to the Creative Commons licence, and indicate if changes were made. The images or other third party material in this article are included in the article's Creative Commons licence, unless indicated otherwise in a credit line to the material. If material is not included in the article's Creative Commons licence and your intended use is not permitted by statutory regulation or exceeds the permitted use, you will need to obtain permission directly from the copyright holder. To view a copy of this licence, visit http://creativecommons.org/licenses/by/4.0/.
